# Synthesis, preclinical evaluation and radiation dosimetry of a dual targeting PET tracer [^68^Ga]Ga-FAPI-RGD

**DOI:** 10.7150/thno.79144

**Published:** 2022-10-09

**Authors:** Jie Zang, Xuejun Wen, Rong Lin, Xinying Zeng, Chao Wang, Mengqi Shi, Xueyuan Zeng, Jiaying Zhang, Xiaoming Wu, Xianzhong Zhang, Weibing Miao, Pengfei Xu, Zhide Guo, Jingjing Zhang, Xiaoyuan Chen

**Affiliations:** 1 Department of Nuclear Medicine, the First Affiliated Hospital, Fujian Medical University, No. 20 Chazhong Road, Taijiang District, Fuzhou 350005, Fujian Province, China.; 2 State Key Laboratory of Molecular Vaccinology and Molecular Diagnostics & Center for Molecular Imaging and Translational Medicine, School of Public Health, Xiamen University, 4221-116 Xiang'An South Rd, Xiamen 361102, China.; 3 Departments of Diagnostic Radiology, Yong Loo Lin School of Medicine, National University of Singapore, Singapore, 119074, Singapore.; 4 Nanomedicine Translational Research Program, NUS Center for Nanomedicine, Yong Loo Lin School of Medicine, National University of Singapore, Singapore 117597, Singapore.; 5 College of Nuclear Science and Technology, Harbin Engineering University, Harbin 150001, China.; 6 Fujian Key Laboratory of Precision Medicine for Cancer, the First Affiliated Hospital, Fujian Medical University, Fuzhou 350005, Fujian Province, China.; 7 Institute of Clinical Pharmacy & Pharmacology, Jining First People's Hospital, Jining Medical University, Jining 272000, China.; 8 Clinical Imaging Research Centre, Centre for Translational Medicine, Yong Loo Lin School of Medicine, National University of Singapore, Singapore 117599, Singapore.; 9 Departments of Surgery, Chemical and Biomolecular Engineering, and Biomedical Engineering, College of Design and Engineering, National University of Singapore, Singapore 117597, Singapore.; 10 Institute of Molecular and Cell Biology, Agency for Science, Technology, and Research (A*STAR), 61 Biopolis Drive, Proteos, Singapore, 138673, Singapore.

**Keywords:** FAPI-RGD, heterodimer, fibroblast activation protein, integrin αvβ3, ^68^Ga

## Abstract

To enhance tumor uptake and retention, we designed and developed bi-specific heterodimeric radiotracers targeting both FAP and αvβ3, [^68^Ga]Ga-FAPI-RGD. The present study aimed to evaluate the specificity, pharmacokinetics, and dosimetry of [^68^Ga]Ga-FAPI-RGD by preclinical and preliminary clinical studies.

**Methods:** FAPI-RGD was designed and synthesized with the quinoline-based FAPI-02 and the cyclic RGDfK peptide. Preclinical pharmacokinetics were determined in Panc02 xenograft model using microPET and biodistribution experiments. The safety and effective dosimetry of [^68^Ga]Ga-FAPI-RGD was evaluated in 6 cancer patients, and compared with 2-[^18^F]FDG imaging.

**Results:** The [^68^Ga]Ga-FAPI-RGD had good stability in saline for at least 4 h, and showed favorable binding affinity and specificity *in vitro* and *in vivo*. Compared to [^68^Ga]Ga-FAPI-02 and [^68^Ga]Ga-RGDfK, the tumor uptake and retention of [^68^Ga]Ga-FAPI-RGD were very much enhanced than its monomeric counterparts at all the time points examined by microPET imaging. A total of 6 patients with various malignant tumors were prospectively enrolled. The effective dose of [^68^Ga]Ga-FAPI-RGD was 1.94E-02 mSv/MBq. The biodistribution of [^68^Ga]Ga-FAPI-RGD from 0 to 2 h after injection demonstrated rapid and high tumor uptake, prolonged tumor retention, and high tumor-to-background ratios (TBRs) which further increased over time. No significant difference in mean SUVmax of [^68^Ga]Ga-FAPI-RGD and 2-[^18^F]FDG was present in primary tumors (8.9±3.2 *vs.* 10.3 ± 6.9; p = 0.459).

**Conclusion:** The dual targeting PET tracer [^68^Ga]Ga-FAPI-RGD showed significantly improved tumor uptake and retention, as well as cleaner background over ^68^Ga-labeled FAPI and RGD monospecific tracers. The first-in-human biodistribution study showed high TBRs over time, suggesting high diagnostic performance and favorable tracer kinetics for potential therapeutic applications.

## Introduction

Fibroblast activation protein (FAP), a type II transmembrane serine protease, is highly expressed in cancer-associated fibroblasts (CAFs) and critically associated with tumor growth, invasion, metastasis, immunosuppression and prognosis 1. In recent years, a variety of quinoline-based FAP inhibitors (FAPIs) have been developed, such as FAPI-02, FAPI-04 and FAPI-46 2, 3. FAPI PET/CT demonstrated advantages over 2-[^18^F]FDG PET/CT for the diagnosis of primary and metastatic lesions of various types of cancer, in terms of uptake, sensitivity, favorable imaging contrast, and ultimately higher tumor detection rate 4, 5. While FAPI grants a wider spectrum of tumor targeting potential compared to conventional radiotracers that recognize tumor cell-specific biomarkers, for example somatostatin receptor subtype-2 targeting DOTATATE and prostate specific membrane antigen targeting PSMA-617; it can be a double-edged sword. High expression of FAP also occurs in chronic inflammation, fibrosis, arthritis, atherosclerotic plaques and cardiac fibrosis 6-9, which results in a compromised sensitivity/selectivity in distinguishing cancer from other FAP-positive diseases, such as chronic inflammation and fibrosis. In addition, short tumor retention time of FAPI-02, FAPI-04 and FAPI-46, although fine for imaging purpose, is suboptimal for radionuclide therapy. Therefore, modifications to FAPI are necessary to confer enhanced tumor-targeting sensitivity as well as extended tumor retention for therapeutic applications.

Integrin α_v_β_3_, which is significantly upregulated in angiogenic endothelial cells and most types of tumor cells but absent in resting endothelial cells of normal tissues, plays an important role in regulating tumor growth, angiogenesis, local invasiveness, and metastatic potential 10. These characteristics make α_v_β_3_ a suitable target for tumor imaging and therapy. The radiolabeled cyclic arginine-glycine-aspartate (RGD) peptides, with integrin α_v_β_3_ binding specificity, have been recognized as potent molecular agents for imaging angiogenesis and tumor 11-13. However, RGD-based radiotracers, including multimeric RGD peptides that were described to have enhanced integrin-targeting efficiency 14-20, had only moderate tumor uptake 21.

**Figure 1:**
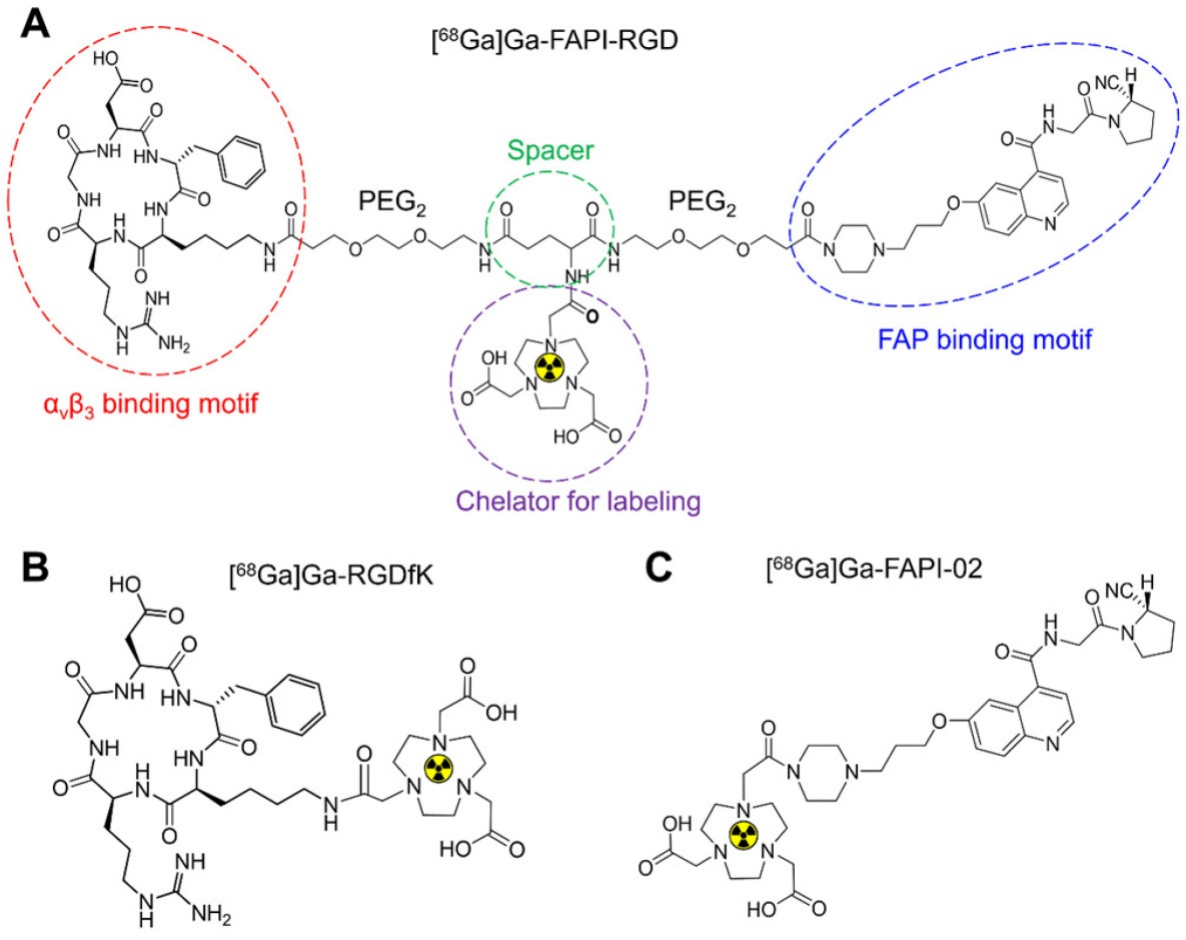
Chemical structures of [^68^Ga]Ga-FAPI-RGD (**A**), [^68^Ga]Ga-RGDfK (**B**) and [^68^Ga]Ga-FAPI-02 (**C**).

Inspired by concept of heterodimeric PET probe that binds to two different targets of tumors, namely gastrin-releasing peptide receptor (GRPR) and integrin α_v_β_3_ 21, we intended to design and develop a heterodimer FAPI-RGD that targets FAP and integrin α_v_β_3_. The dual FAP- and α_v_β_3_-targeting structure of FAPI-RGD heterodimer is illustrated in Figure 1. It is hypothesized that FAPI-RGD would not only confer extra tumor specificity over other diseases, which is rationalized by the expression of both FAP and integrin α_v_β_3_ in a variety of tumors; but also outperform its monomeric counterparts, with regard to tumor uptake and tumor retention.

In this work, FAPI-RGD was designed and synthesized with the following major components: a quinoline-based FAPI-02 for targeting FAP; a cyclic RGDfK peptide for targeting α_v_β_3_; a 1,4,7-triazacyclononanetriacetic acid (NOTA) group for radionuclide labeling; and poly (ethylene glycol) linker. [^68^Ga]Ga-FAPI-RGD was compared with its monomeric counterparts *in vitro* and *in vivo*. With preclinical data consolidating the dual targeting concept of FAPI-RGD, its tumor imaging potential, safety and dosimetry in cancer patients were preliminarily evaluated.

## Materials and methods

### General

All chemicals, reagents and solvents for heterodimer synthesis and downstream preclinical analysis were of analytical grade. ^68^Ga was eluted from ^68^Ge/^68^Ga generator on site. Radioactivity was measured using a CRC-25R Dose Calibrator (CAPIN-TEC Inc., USA) and γ-counter (WIZARD 2480; Perkin-Elmer, USA). Radiolabeling efficiency and radiochemical purity were tested using a radio thin layer chromatography (radio-TLC; BioScan, USA) and radio high performance liquid chromatography (HPLC) was performed using Dionex Ulti-Mate 3000 (Thermo Scientific, USA) with an Elysia Raytest Gabi Star γ-radiation detector. MicroPET/CT scan was performed using an Inveon scanner (Siemens PET, USA). Patients imaging was performed on a PET/CT scanner (Biograph mCT64, Siemens Healthcare).

### Chemistry and Radiochemistry

A schematic demonstration of detailed chemical synthesis procedures is elaborated in the Supplementary Materials. Radiolabeling of ^68^Ga was performed by incubation with 50 μg FAPI-RGD in NaOAc buffer solution (pH = 4.5) at 95 ℃ for 10 min. The product was purified by C18 column extraction. The radiochemical purity and stability of [^68^Ga]Ga-FAPI-RGD were determined by radio-HPLC.

### Cell Culture and Uptake Assays

Panc02 cell line was purchased from the American Type Culture Collection (ATCC), and cultured in Dulbecco modified Eagle medium (DMEM) containing 10% (v/v) fetal bovine serum (FBS) at 37 °C with 5% CO_2_. Panc02 cells were seeded in 24-well plates with 2 × 10^5^ cells per well. After 24 h, 37 kBq of [^68^Ga]Ga-FAPI-RGD, [^68^Ga]Ga-FAPI-02 and [^68^Ga]Ga-RGDfK, respectively, were diluted in 0.5 mL medium, added into adherent cells, and cultured for 10, 30, 60, 90, and 120 min at 37 °C. Then, the medium was removed and the cells were washed twice with cold PBS (pH 7.4) and subsequently lysed with 0.5 mL of NaOH (1 M). In the blocking studies, excess amount of unlabeled FAPI-RGD, FAPI-02, RGDfK, FAPI-02+RGDfK (10 μg/well) were added as inhibitors into the cells along with [^68^Ga]Ga-FAPI-RGD. After 1 h, the cells were treated in the same way. Cell lysates was collected and the radioactivity was determined using a γ counter.

### Animal Models

All animal studies were approved by the Animal Care and Use Committee of Xiamen University and carried out in compliance with the national laws related to the conduct of animal experiments. Panc02 tumor model was generated by subcutaneous injection of 5 × 10^6^ tumor cells suspended in 100 μL PBS, into the upper limb flank of C57BL/6 mice (Beijing Vital River Laboratory Animal Technology Co., Ltd.). The tumor mice were subjected to microPET and biodistributions studies when the tumor volume reached 200-300 mm^3^.

### MicroPET Imaging

PET scans were performed by using Inveon small-animal PET scanner (Siemens Preclinical Solution). About 7.4 MBq of [^68^Ga]Ga-FAPI-RGD was given to Panc02 tumor xenografted mice through tail vein injection for the 10-min static PET imaging. At 0.5, 1, 2 and 4 h post-injection (p.i.), mice were anaesthetized and placed on imaging chamber of PET/CT scanner for image acquisition. For comparison, [^68^Ga]Ga-FAPI-02 and [^68^Ga]Ga-RGDfK were intravenously injected to Panc02 tumor mice and PET imaging was performed at 0.5, 1 and 2 h p.i. The blocking study was performed by injecting unlabeled c(RGDfK), FAPI-02, or RGDfK+FAPI-02, before administering [^68^Ga]Ga-FAPI-RGD. PET images were reconstructed using three-dimensional ordered-subset expectation-maximization (3D OSEM) algorithm and with a Maximum a Posteriori (MAP) method and analyzed through drawing regions of interest (ROIs).

### Biodistribution Study

Panc02 tumor mice were randomly divided into 4 groups (n = 4/group) and injected with [^68^Ga]Ga-FAPI-RGD (740 kBq in 100 µL saline), and sacrificed at 0.5, 1, 2 and 4 h p.i. Tissue and organs of interest were collected, wet weighted and activity measured by a γ-counter. Meanwhile, [^68^Ga]Ga-FAPI-02 and [^68^Ga]Ga-c(RGDfK) were injected into Panc02 tumor model 1 h p.i. for biodistribution studies as the control groups. The results were calculated as percentage of injected dose per gram (%ID/g).

### Patients

This study was approved by the institutional review board of the First Affiliated Hospital, Fujian Medical University and registered at ClinicalTrials.gov (NCT05515783). All patients signed an informed consent form before participation and all procedures were conducted in accordance with the Declaration of Helsinki. The inclusion criteria were as follows: (1) patients with newly diagnosed cancer and no treatment before [^68^Ga]Ga-FAPI-RGD PET/CT scanning; (2) primary malignancies confirmed by pathology. The exclusion criteria were patients: (1) who suffered from severe hepatic and renal insufficiency; (2) pregnant or lactational women.

### PET/CT Image Acquisition

All patients underwent [^68^Ga]Ga-FAPI-RGD PET/CT and 2-[^18^F]FDG PET scanning using a dedicated PET/CT scanner (Biograph mCT64, Siemens Healthcare).

**[^68^Ga]Ga-FAPI-RGD PET/CT** The CT scans were performed with a tube voltage of 120 kV, an effective tube current of 70-120 mA (Care Dose 4D), and a slice thickness of 3 mm. Serial whole-body dynamic PET scans were immediately performed after the CT scan in 3D acquisition mode (matrix: 200 × 200). The whole body (from the top of skull to the middle of femur) of each patient was covered by 6 bed positions. The acquisition duration was 30 s/bed position at 3 min after injection; 1 min/bed position at 15 min after injection; and 2 min/bed position at 30, 60, and 120 min after injection. PET data were reconstructed iteratively (2 iterations and 21 subsets) with CT data for attenuation correction, and the PET/CT images were then co-registered and displayed using dedicated software (TrueD software, Siemens).

**2-[^18^F]FDG PET/CT** All patients were fast at least 6 h to keep and images were obtained at 60-80 min after the intravenous injection of 2-[^18^F]FDG (3.70 MBq [0.10 mCi]/kg). 2-[^18^F]FDG was purchased from Fuzhou PET-Tracer Co. Ltd, and the radiochemical purity was more than 95%.

Any drug-related side effects were recorded and the vital parameters of the patients were observed for 1 week.

### Radiation Dosimetry Estimate

Organ delineation and activity accumulation at each imaging time point was determined using Hybrid-Dosimetry software (Hermes Medical Solutions, Sweden). Time-activity curve fitting and subsequent dose calculation was performed using OLINDA/EXM, version 2.2.0. Kidneys, liver, spleen, urinary bladder content, L2-L4 lumbar vertebrae for red marrow dosimetry, heart content, lung, brain, pancreas, thyroid, salivary glands and stomach contents were included as source organs. Red bone marrow dosimetry was done by image-based 3D volumetric analysis of L2-L4 vertebrae, which is considered to constitute 6.7% of the total bone marrow 22. The ROIs for the above organs were drawn from the examination that had the best organ delineation and then were copied onto all other time points to calculate the time-activity curves. The mono- or bi-exponential curve fitting parameters were applied to derive the best curve fit for the residence time of activity in the source organ. Once all the organ residence times were derived from the kinetic input model, they were entered in the adult female or male model data to derive the absorbed doses to all the organs, including the whole-body effective dose, and generated in mSv/MBq and rem/mCi. The time-activity graphs of various organs were generated using GraphPad Prism software.

### Data Analysis and Statistics

Calculations were performed using SPSS (IBM SPSS Statistics for Windows, version 21.0). P values of less than 0.05 were accepted as statistically significant. All quantitative data were expressed as the mean ± SD.

## Results

### Chemistry and Radiochemistry

The chemical structure of synthesized FAPI-RGD is shown in **Figure 1**. FAPI-RGD and its intermediate products were characterized by TOF-MS and HPLC (**Figure S1-9**). The radiolabeling yield and radiochemical purity of [^68^Ga]Ga-FAPI-RGD were consistently over 99%. Radio-HPLC results showed that the synthesized [^68^Ga]Ga-FAPI-RGD had good stability in saline for at least 4 h. The radiochemistry purity was over 99% within 4 h.

### Cellular Uptake and Blocking Assays

**Figure 2:**
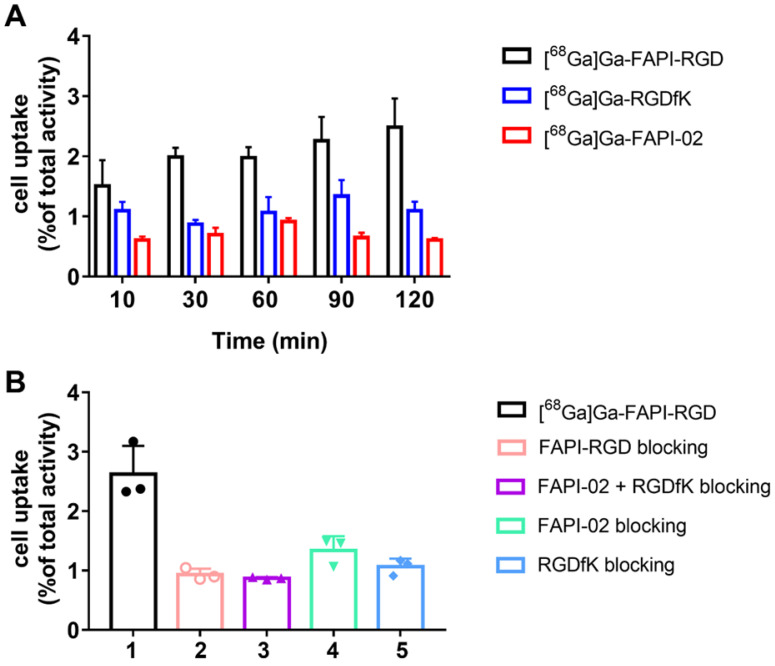
** (A)** Cellular uptake assay of [^68^Ga]Ga-FAPI-RGD, [^68^Ga]Ga-RGDfK and [^68^Ga]Ga-FAPI-02 (n = 4/group) on Panc02 tumor cells. **(B)** Blocking assays of [^68^Ga]Ga-FAPI-RGD on Panc02 tumor cells by unlabeled FAPI-RGD, FAPI-02+RGDfK, FAPI-02 and RGDfK (n = 3/group).

In Panc02 tumor cells, [^68^Ga]Ga-FAPI-RGD had a gradually increased uptake over time until 120 min. As shown in **Figure 2A**, the highest cellular uptake of [^68^Ga]Ga-FAPI-RGD was observed (2.48 ± 0.44%) after incubation for 120 min. For comparison, [^68^Ga]Ga-FAPI-02 and [^68^Ga]Ga-c(RGDfK) incubated with Panc02 tumor cells for the same amount of time. The cellular uptake values were less than [^68^Ga]Ga-FAPI-RGD at all the time points. Cell uptake of [^68^Ga]Ga-FAPI-RGD could be blocked by addition of unlabeled FAPI-RGD, FAPI-02, RGDfK, or FAPI-02 + RGDfK to some extent. As exhibited in **Figure 2B**, after incubation for 120 min, cell uptake of [^68^Ga]Ga-FAPI-RGD was decreased to 0.93 ± 0.08% in FAPI-RGD blocking assay; to 1.34 ± 0.21% in FAPI-02 blocking assay; to 1.06 ± 0.12% in RGDfK blocking assay; and to 0.87 ± 0.02% in FAPI-02 + RGDfK blocking assay.

### MicroPET Imaging

**Figure 3:**
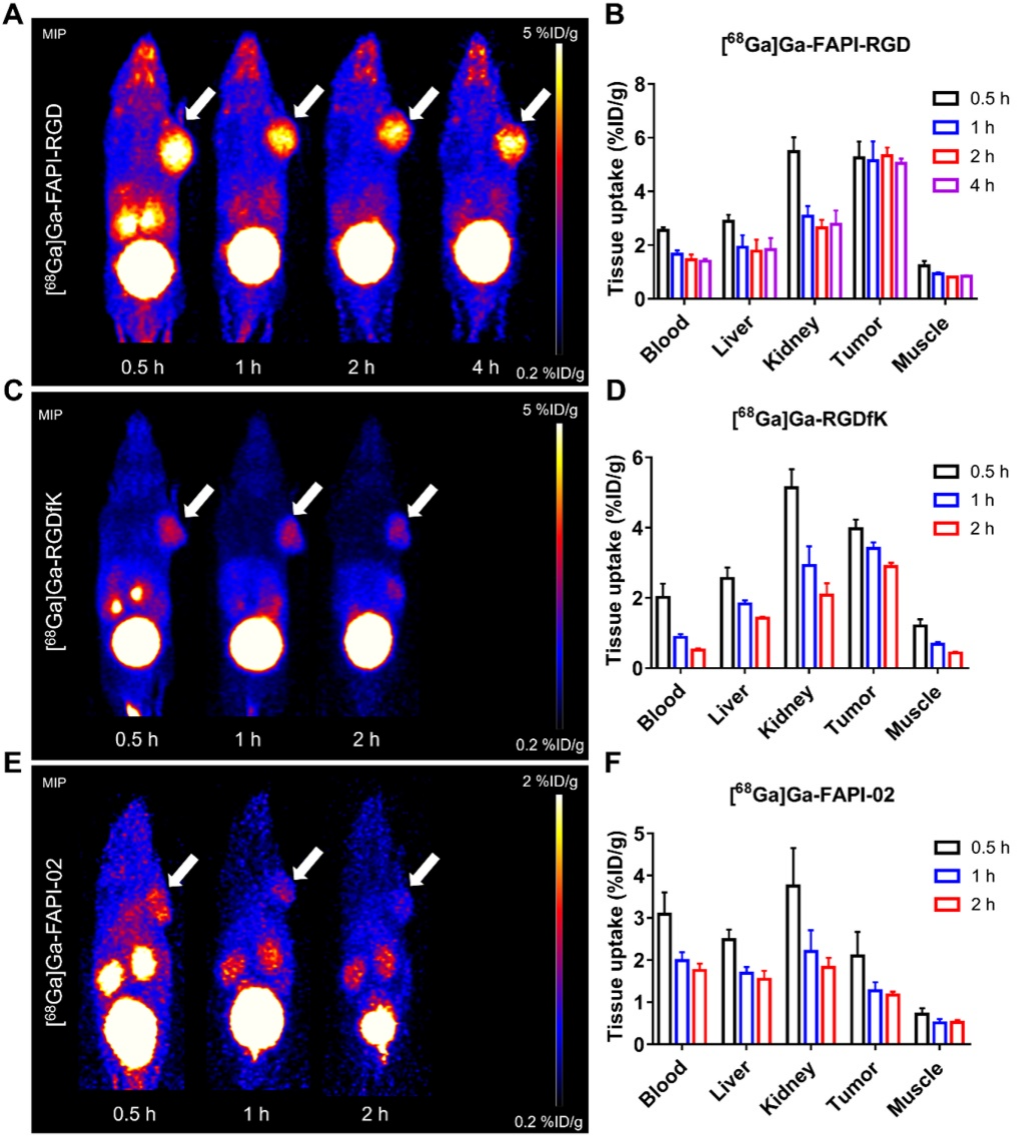
** (A)** PET images of [^68^Ga]Ga-FAPI-RGD in Panc02 tumor-bearing mice at 0.5, 1, 2, and 4 h p.i.; in comparison with PET images of [^68^Ga]Ga-FAPI-02 **(C)** and [^68^Ga]Ga-RGDfK **(E)**; PET images shown are 10-min static scans of a single mouse, which is representative of the 3 mice tested in each group; **(B, D and F)** The uptakes of [^68^Ga]Ga-labeled FAPI-RGD, FAPI-02, and RGDfK at different time points.

Static microPET scans were performed on Panc02 tumor bearing mice (n = 3), and representative whole-body scan images at different time points after injection of [^68^Ga]Ga-FAPI-RGD, [^68^Ga]Ga-FAPI-02, or [^68^Ga]Ga-RGDfK are shown in **Figure 3**. The tumor uptake of [^68^Ga]Ga-FAPI-RGD was significantly higher than normal tissues (**Figure 3A**), tumor uptake values were calculated to be 5.25 ± 0.54, 5.13 ± 0.65, 5.33 ± 0.27, and 5.04 ± 0.16 %ID/g at 0.5, 1, 2 and 4 h after injection. While, normal tissue uptakes of [^68^Ga]Ga-FAPI-RGD were lower than tumor, especially at 1, 2 and 4 h p.i. (blood uptake was 1.40 ± 0.08 %ID/g, liver uptake was 1.83 ± 0.39 %ID/g, kidney uptake was 2.78 ± 0.46 %ID/g at 4 h) (**Figure 3B**). By contrast, PET imaging of [^68^Ga]Ga-RGDfK was shown in **Figure 3C**, tumor signal was obvious and uptake values were 3.96 ± 0.24, 3.40 ± 0.16, and 2.89 ± 0.09 %ID/g at 0.5, 1 and 2 h p.i. (**Figure 3D**). For [^68^Ga]Ga-FAPI-02, moderate tumor uptakes were observed (**Figure 3E**). The tumor uptakes were 2.09 ± 0.51, 1.26 ± 0.18, and 1.16 ± 0.07 %ID/g at 0.5, 1 and 2 h after injection (**Figure 3E**). The increased tumor uptake and prolonged tumor retention time of the heterodimeric tracer was clearly demonstrated by PET imaging.

**Figure 4:**
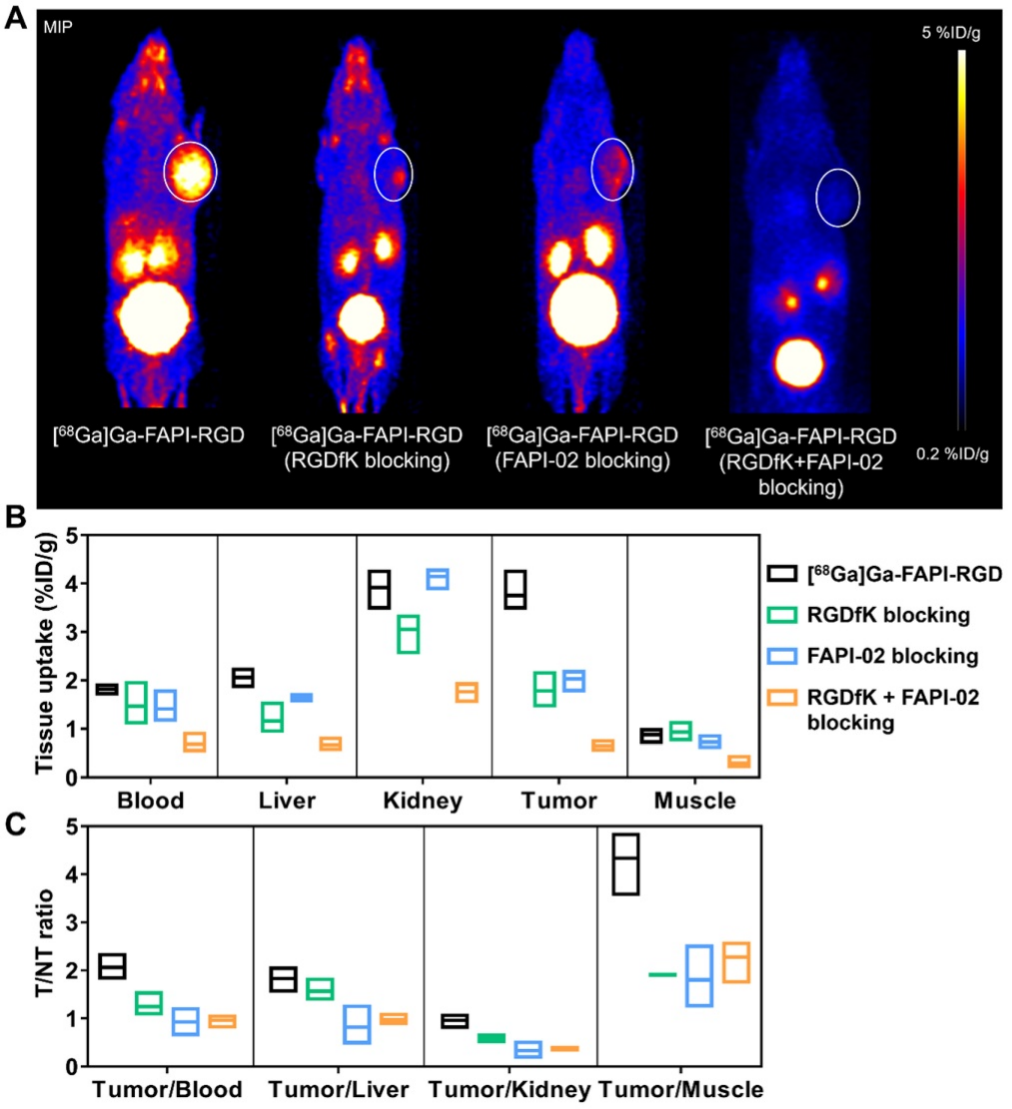
** (A)** Representative PET images of [^68^Ga]Ga-FAPI-RGD at 0.5 h p.i., and blocking with unlabeled RGDfK, FAPI-02, or RGDfK + FAPI-02, respectively, in Panc02 tumor mice at the same time points. The quantification results **(B)** and T/NT ratios **(C)** of PET imaging of [^68^Ga]Ga-FAPI-RGD with or without blocking.

To evaluate the targeting specificity, unlabeled RGDfK, FAPI-02, or RGDfK + FAPI-02 were co-administered with [^68^Ga]Ga-FAPI-RGD. The tumor uptake was greatly inhibited by blocking with unlabeled RGDfK, FAPI-02, or RGDfK + FAPI-02 in Panc02 tumor mice (**Figure 4A**). For RGDfK + FAPI-02 blocking, tumor uptake not only decreased significantly than control group, but also lower than monomeric RGDfK or FAPI-02 blocking groups. The tissue uptake values of PET imaging with or without inhibitors are presented in **Figures 4B-C**. The significant differences were observed for tumor uptake and tumor/muscle ratios.

### Biodistribution Study

**Figure 5:**
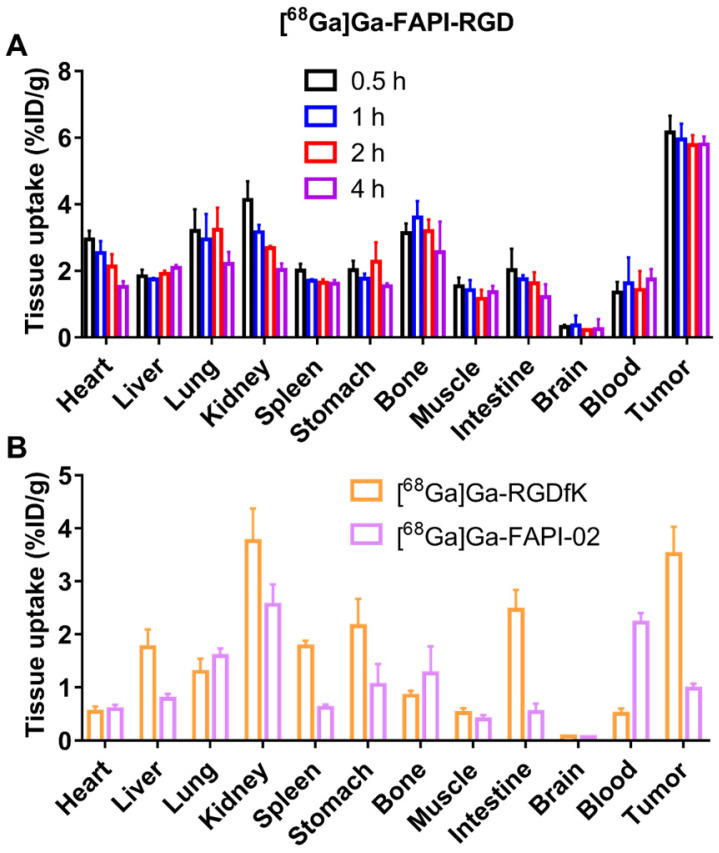
** (A)** The biodistribution of [^68^Ga]Ga-FAPI-RGD in Panc02 tumor mice at 0.5, 1, 2 and 4 h p.i. (n = 4/group); **(B)** The biodistribution of [^68^Ga]Ga-FAPI-02 and [^68^Ga]Ga-RGDfK in Panc02 tumor model at 1 h p.i. (n = 4/group).

To further evaluate the pharmacokinetics properties *in vivo*, biodistribution studies using the Panc02 tumor mice were performed. As shown in **Figure 5A**, the tumor uptakes of [^68^Ga]Ga-FAPI-RGD were calculated to 6.16 ± 0.46, 5.95 ± 0.43, 5.78 ± 0.28, and 5.79 ± 0.22 %ID/g at 0.5, 1, 2 and 4 h p.i., respectively. As consistent with the results of PET imaging, the major organ uptakes of [^68^Ga]Ga-FAPI-RGD were lower than that of tumor at all the time points examined. For comparison, biodistribution studies of [^68^Ga]Ga-RGDfK and [^68^Ga]Ga-FAPI-02 were conducted in Panc02 tumor mice at 1 h p.i. (**Figure 5B**). The tumor uptake of [^68^Ga]Ga-RGDfK was 3.51 ± 0.47 %ID/g, and that of [^68^Ga]Ga-FAPI-02 was 0.97 ± 0.09 %ID/g at 1 h p.i., both of which were lower than that of [^68^Ga]Ga-FAPI-RGD.

### Patient characteristics and safety

A total of 6 patients (3 males, 3 females; median age, 64 years; range, 57-82 years) with various malignant tumors were prospectively enrolled, including 2 patients with lung cancer, 1 patient with nasopharyngeal cancer, 1 patient with breast cancer, 1 patient with renal carcinoma, and 1 patient with oral carcinoma. The injected activity of [^68^Ga]Ga-FAPI-RGD ranged from 173 to 247 MBq (4.7-6.7 mCi). All patients tolerated the examination well. Vital parameters remained stable and no patient reported any new symptoms during the observation period.

### Patient Biodistribution

**Figure 6:**
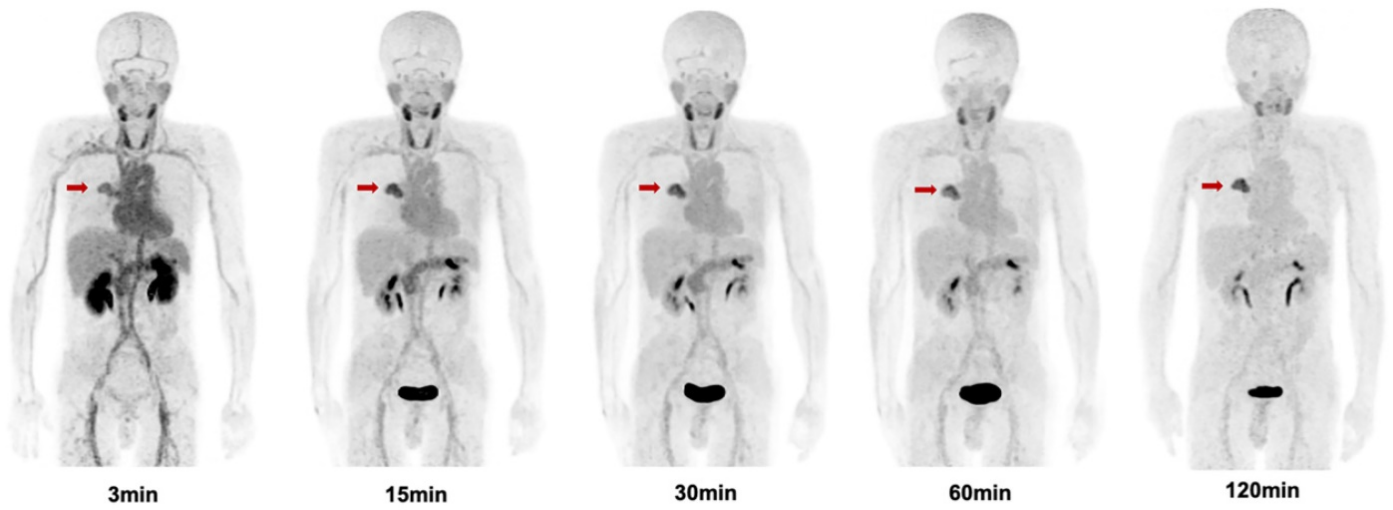
Maximum intensity projection (MIP) images of [^68^Ga]Ga-FAPI-RGD at 3,15, 30, 60, and 120 min post injection in a patient with lung cancer (Lung cancer lesions are indicated by red arrows).

**Figure 7:**
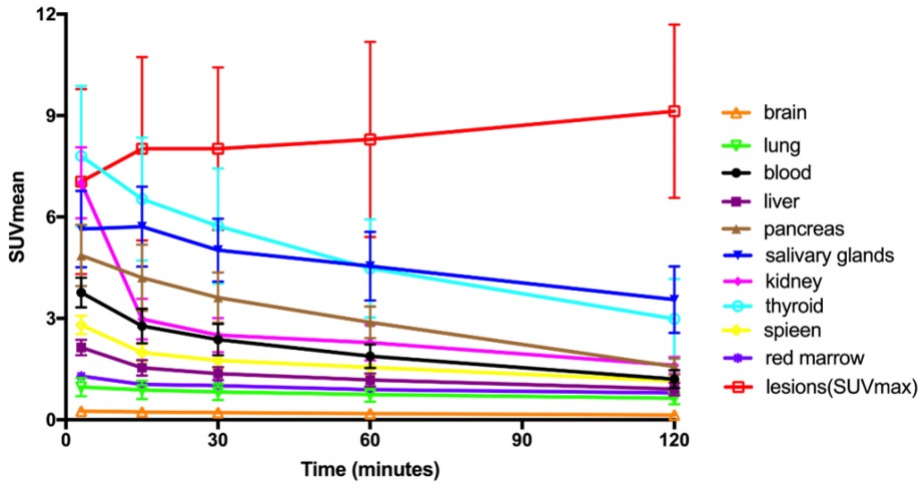
Time-activity curves of [^68^Ga]Ga-FAPI-RGD at different time points following tracer injection.

The biodistribution of [^68^Ga]Ga-FAPI-RGD in normal organs and tumor is presented in **Figure 6** and illustrated as time-dependent maximum-intensity projections in **Figure 7**. The physiological biodistribution of [^68^Ga]Ga-FAPI-RGD involved the thyroid glands, salivary glands, pancreas, kidneys, liver, heart content, spleen, and urinary bladder. The SUVmax of normal organs decreased in all patients over time, except for the bladder. [^68^Ga]Ga-FAPI-RGD PET/CT demonstrated intense radioactivity in the urinary tract, indicating that the kidneys were the main excretory organs. The highest average normal organ SUVmean at all time points was observed in the thyroid glands, decreasing from an average SUVmean of 7.8 at 3 min to 3.0 by 2.0 h (decline of 62.5%).

Tracer uptake in the tumor was rapid and showed a steady increase in SUV values with an average SUVmax of 7.0 at 3 min, 8.0 at 15 min, 8.0 at 30 min, 8.3 at 60 min and 9.1 at 2 h, respectively. Meanwhile, the TBRs increased significantly to a much higher level with time (with exception of the gallbladder TBR) due to the SUVmax of the organs decreased over time. The TBR (blood) was 2.7 at 15 min, 4.1 at 60 min, and 6.9 at 2 h. The TBR (liver) was 5.0 at 15 min, 6.7 at 60 min, and 9.7 at 2 h, higher than TBR (blood). The lesions could be clearly visualized at the early 15-min scan, which became clearer at the 1-h and 2-h scans.

### Radiation Dosimetry

The dosimetry reports for 6 patients are shown in Table 1. The organ with the highest effective dose was the urinary bladder wall (2.26E-01 mSv/MBq), followed by the thyroid (3.31E-02 mSv/MBq), Kidneys (3.24E-02 mSv/MBq), and pancreas (3.06E-02 mSv/MBq). The effective dose of [^68^Ga]Ga-FAPI-RGD was 1.94E-02 mSv/MBq. Thus, for administration of 185 MBq (5.0 mCi) of [^68^Ga]Ga-FAPI-RGD, the total body effective dose was 3.6 mSv.

### Comparison between [^68^Ga]Ga-FAPI-RGD and 2-[^18^F]FDG in tumor uptake

**Figure 8:**
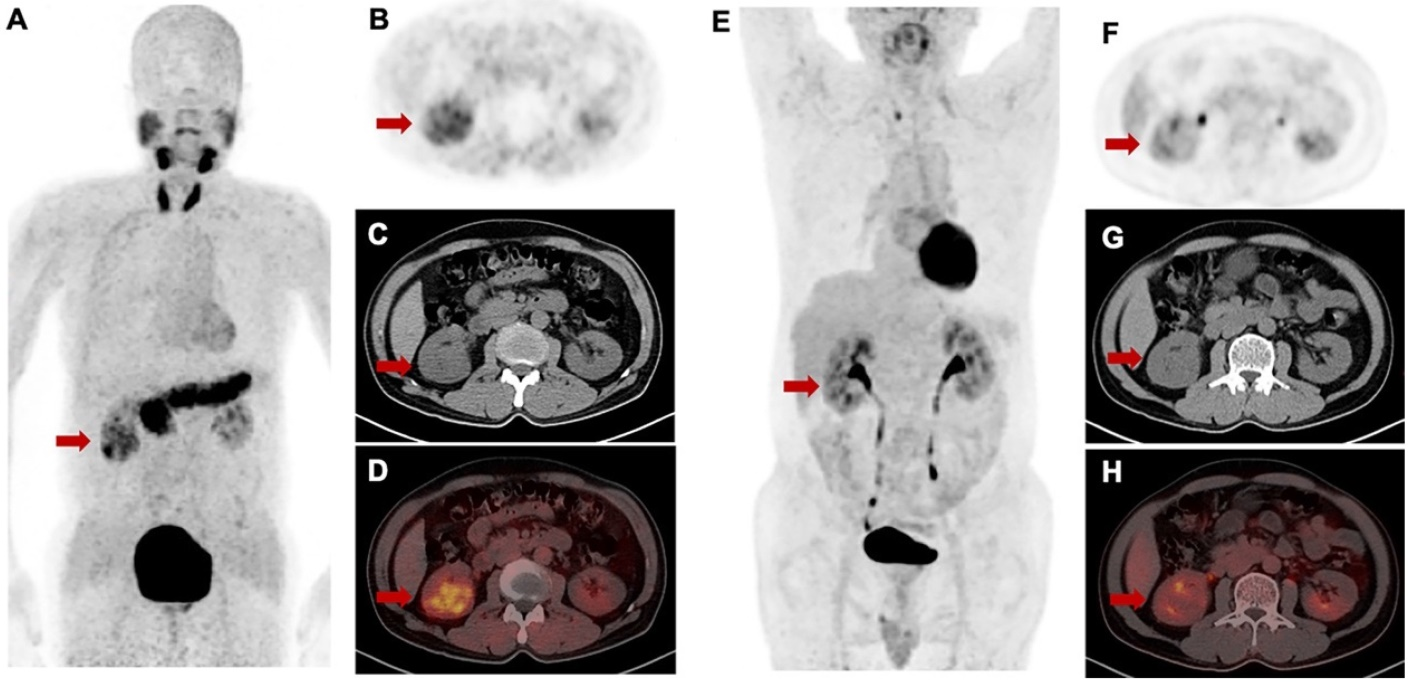
Intra-individual comparison of [^68^Ga]Ga-FAPI-RGD **(A-D)** and 2-[^18^F]FDG **(E-H)** in a patient with renal cell carcinoma. [^68^Ga]Ga-FAPI-RGD showed intense uptake in the primary lesion (SUVmax = 5.1), while there was only slight uptake on 2-[^18^F]FDG PET/CT (SUVmax = 2.7).

For [^68^Ga]Ga-FAPI-RGD PET/CT, only PET/CT datasets acquired approximately 1 h after injection were used for analysis. No significant difference in mean SUVmax of [^68^Ga]Ga-FAPI-RGD and 2-[^18^F]FDG was present in primary tumors (8.9 ± 3.2 *vs.* 10.3 ± 6.9; p = 0.459). However, marked differences in tumors uptakes between [^68^Ga]Ga-FAPI-RGD and 2-[^18^F]FDG were observed in the patient with renal carcinoma. The tumor lesions with low uptake on 2-[^18^F]FDG PET but high uptake on [^68^Ga]Ga-FAPI-RGD scan is shown in **Figure 8**. Among the six patients, three patients had metastatic foci. The number and SUVmax of metastasis were not significantly different between [^68^Ga]Ga-FAPI-RGD and 2-[^18^F]FDG (n = 70 *vs.* n = 69, p = 0.986; for SUVmax, 6.5 ± 2.6 *vs.* 7.6 ± 5.3, p = 0.373).

## Discussion

As part of clinical practice of theranostics, molecular imaging is used as a non-invasive and quantitative measure for tumor visualization, characterization and prognosis, using specific tracers. Thus far, clinical molecular imaging has been largely nuclear medicine based on the forms of PET and SPECT 23. Over the last few decades, many radiotracers have been explored for PET imaging, some of which have been applied clinically and approved by FDA 24-26. Compared to those monomeric radiotracers developed, homodimeric radiotracers have been proven to have enhanced tumor uptake and retention 14-20. However, due to the tumor heterogeneity and complexity, the conventional monotargating radiotracers may have compromised diagnosis sensitivity and specificity.

FAP and integrin α_v_β_3_ are two highly expressed targets in tumor stroma and angiogenic endothelial cells. To exploit the favorable properties of heterodimers, we designed and developed bi-specific heterodimeric radiotracers targeting both FAP and α_v_β_3_ for [^68^Ga]Ga-based PET imaging in multiple tumor types. The cellular uptake assay demonstrated that the high uptake of novel dual targeting tracer [^68^Ga]Ga-FAPI-RGD can only be completely blocked in the presence of both monomeric counterparts, suggesting the favorable binding affinity and specificity towards both FAP and α_v_β_3_.

The PET imaging potential of [^68^Ga]Ga-FAPI-RGD was tested in Panc02 xenograft model. Compared to [^68^Ga]Ga-FAPI-02 and [^68^Ga]Ga-RGDfK, the tumor uptake and retention of [^68^Ga]Ga-FAPI-RGD were very much enhanced than both of its monomeric tracers at all the time points examined. The pharmacokinetics were also improved to a great extent. This decreased tumor uptake in normal organs and enhanced uptake in tumor confer [^68^Ga]Ga-FAPI-RGD with higher tumor-to-normal ratios when compared with [^68^Ga]Ga-FAPI-02 and [^68^Ga]Ga-RGDfK. In the blocking experiments, both non-radiolabeled FAPI-02 inhibitors and RGDfK peptides showed partial inhibition toward the uptake of [^68^Ga]Ga-FAPI-RGD in Panc02 tumor, proving the bi-specificity of the designed tracer. In contrast, the FAPI-02 and RGDfK double blocking could further reduce the tumor uptake of [^68^Ga]Ga-FAPI-RGD to an almost undetectable level, substantiating a synergistic effect of both monomeric counterparts in tumor targeting. Obviously, one additional advantage of our novel FAPI-RGD heterodimer is its ability to target tumors in the presence of only one of the targets in tumors. The overexpression of either target in tumors is sufficient for [^68^Ga]Ga-FAPI-RGD to detect with great sensitivity.

With high expression of FAP and/or α_v_β_3_ being described in multiple tumor types, our preclinical success prompted us to conduct the preliminary first-in-human study, which suggests that [^68^Ga]Ga-FAPI-RGD can be used for visualization of a wide spectrum of tumors, including those of FAP^-^/α_v_β_3_^+^, FAP^+^/ α_v_β_3_^-^, and FAP^+^/α_v_β_3_^+^.

Herein we described the biodistribution of [^68^Ga]Ga-FAPI-RGD and its estimated radiation dose deposition in the organs of 6 cancer patients whom underwent [^68^Ga]Ga-FAPI-RGD PET/CT imaging at 5 time points. The effective dose of [^68^Ga]Ga-FAPI-RGD (1.94 mSv/100 MBq) is comparable to that of [^68^Ga]Ga-FAPI tracers (0.8-1.8 mSv/100 MBq) 27-29 and [^68^Ga]Ga-RGD tracers (2.1-2.5 mSv/100 MBq) 30-32.

The biodistribution of [^68^Ga]Ga-FAPI-RGD from 0 to 2 h after injection demonstrated rapid and high tumor uptake, long tumor retention, and high TBRs which further increased over time. However, high physiologic uptakes in the thyroid and pancreas should be noted. [^68^Ga]Ga-FAPI-RGD was predominantly excreted from urinary system, which is similar to the reports for the other [^68^Ga]Ga-FAPI tracers. The favorable pharmacokinetics and prolonged tumor uptake warrant future development of suitably labeled FAPI-RGD derivatives for radioligand therapy.

Preliminary comparison of [^68^Ga]Ga-FAPI-RGD and 2-[^18^F]FDG in the 6 patients showed no significant difference in mean SUVmax in primary tumors and metastasis. However, we observed high [^68^Ga]Ga-FAPI-RGD uptake and low 2-[^18^F]FDG uptake in one patient with renal carcinoma. This finding should be confirmed by in more patients in future studies.

There are still exist some limitations of the present study. This clinical analysis was preliminary, with limited number of patients and no healthy subjects involved. Further investigations are warranted in a large cohort of patients to evaluate the [^68^Ga]Ga-FAPI-RGD uptake in different cancers, with thorough comparison made with its monospecific counterparts, FAPI and/or RGD tracers.

## Conclusion

The newly synthesized heterodimeric PET radiotracer [^68^Ga]Ga-FAPI-RGD, with dual FAP and integrin α_v_β_3_ targeting ability, showed significantly improved tumor uptake, prolonged tumor retention, tumor-targeting efficiency and pharmacokinetics compared with [^68^Ga]Ga-labeled FAPI and RGD monospecific tracers. The first-in-human biodistribution study showed high TBRs over time, suggesting high diagnostic performance and favorable tracer kinetics for potential therapeutic applications.

## Supplementary Material

Supplementary materials and methods, figures.

## Figures and Tables

**Table 1:** Estimated Absorbed Dose After Intravenous Administration of [^68^Ga]Ga-FAPI-RGD (mSv/MBq)

Target Organ	Mean	SD
Adrenals	1.04E-02	5.75E-04
Brain	3.38E-03	6.76E-04
Esophagus	7.55E-03	3.48E-04
Eyes	5.47E-03	4.71E-04
Gallbladder Wall	8.35E-03	2.01E-04
Left colon	7.80E-03	5.34E-04
Small Intestine	8.16E-03	4.98E-04
Stomach Wall	1.20E-02	2.70E-03
Right colon	7.53E-03	4.12E-04
Rectum	1.06E-02	6.43E-04
Heart Wall	2.04E-02	2.80E-03
Kidneys	3.24E-02	7.20E-03
Liver	1.45E-02	3.60E-03
Lungs	2.33E-02	4.41E-03
Pancreas	3.06E-02	1.11E-02
Prostate	1.24E-02	7.97E-04
Salivary Glands	2.09E-02	2.38E-03
Red Marrow	1.45E-02	1.30E-03
Osteogenic Cells	1.07E-02	8.87E-04
Spleen	2.25E-02	9.45E-03
Testes	7.13E-03	4.65E-04
Thymus	7.41E-03	3.27E-04
Thyroid	3.31E-02	7.42E-03
Urinary Bladder Wall	2.26E-01	3.31E-02
Total Body	8.92E-03	4.18E-04
Effective Dose	1.94E-02	1.74E-03
