# Supplementary material for: Synthesis, preclinical evaluation and radiation dosimetry of a dual targeting PET tracer [68Ga]Ga-FAPI-RGD
Source: Theranostics. 2022 Oct 9;12(16):7180–90. doi: 10.7150/thno.79144 (PMC9576616; doi:10.7150/thno.79144)
Supplement: Supplementary file 1 — Supplementary materials and methods, figures. [file thnov12p7180s1.pdf]

Supplementary materials

Synthesis, preclinical evaluation and radiation dosimetry of a dual targeting PET tracer [^{68}Ga]Ga-FAPI-RGD

Materials and characterization

General

All chemical reagents were obtained from the commercial suppliers and used without further purification. MALDITOF-MS spectra were obtained by an AB SCIEX 4700 TOF/TOF System. Analytical high-performance liquid chromatography (HPLC) was done on symmetry C-18 columns from YMC (3 μm, 150 × 4.6 mm i.d.) using 0.1% trifluoroacetic acid (TFA)/H_2O (solvent A) and 0.1% trifluoroacetic acid (TFA) CH_3CN (solvent B) at flow rate of 1 mL/min. Gradient elution was performed as follows: 10% of B, 0-3 min;10-90% of B, 3-15 min; 90% of B, 15-16 min; 90-10% of B, 16-18 min; 10% of B, 18-20 min. The ultraviolet (UV) absorbance was monitored at 254 nm.

Preparation of NOTA-RGD-FAPI (Compound K)

Preparation of Compound B: SM1 was prepared according to our previous work[1]. Then, 2.50 g of SM1, 2.58 g of p-toluenesulfonic acid monohydrate and 25 mL of acetonitrile were added into the reaction bottle, the mixture was reacted at 65 °C for 1 h. When the SM1 reaction was complete as monitored by TLC (methanol: dichloromethane = 5:1), it was evaporated to dryness under reduced pressure at 40 °C. Then 14 mL of DMF and 3.05 g of DIPEA were added into the mixture and stirred at 25 °C, the reaction was numbered as (1). 1.62 g of t-Boc-N-amido-PEG2-acid, 2.60 g of HATU and 10 mL of DMF was added into another reaction bottle, the mixture was reacted at 25 °C for 30 min, the reaction was numbered as (2). Next, the reaction (2) was added to reaction (1), the mixture was reacted for 1h, evaporated to dryness under reduced pressure at 40 °C, and then 50 mL of purified water was added into it. Afterwards, it was extracted twice with DCM, 50 mL each time, combined DCM phases, dried with anhydrous sodium sulfate, filtered and evaporated to dryness to obtain crude product, purified by column chromatography to obtain 1.68g of the target

product. The reaction mixture was concentrated in vacuo and the residue was purified by silica gel chromatography (DCM:MeOH = 50:1 v/v) to give **Compound B** (475 mg, 67% yield). ESI: m/z 710.38 $(M+H)^+$.

Preparation of Compound D: 2.00 g of **Compound B**, 1.61 g of p-toluenesulfonic acid monohydrate, and 20 mL of acetonitrile were added into the reaction bottle, the mixture was reacted at 65 °C for 1 h, and evaporated to dryness under reduced pressure at 40 °C. Then, 20 mL of DMF and 1.83 g of DIPEA were added into the mixture and stirred at 25 °C, the reaction was numbered as (1). 1.43 g of Fmoc-L-glutamic acid 5-tert-butyl ester, 1.29 g of HATU and 20 mL of DMF were added into another reaction bottle, the mixture was reacted at 25 °C for 30 min, the reaction was numbered as (2). Next, the reaction (2) was added to reaction (1), the mixture was reacted for 1 h. Afterwards, it was evaporated to dryness under reduced pressure at 40 °C to obtain crude product, then purified by column chromatography to obtain 1.19 g of **Compound D**. ESI: m/z 1017.51 $(M+H)^+$.

Preparation of Compound G: 1.00 g of c(RGDfk), 0.74 g of t-Boc-N-amido-PEG2-NHS ester, 0.44 g of DIPEA and 20 mL of DMF were added into the reaction bottle, the mixture was reacted at 30 °C for 20 h, and evaporated to dryness under reduced pressure at 40 °C. 10 ml of methanol was added into it and 60 ml of MTBE was dropped into it, after which it solidifies, Then, it was filtered by suction, and vacuum dried at 40 °C for 2 h. The solid, 30 mL of TFA and 1.5 mL of purified water were added into the reaction bottle, the mixture was reacted at 30 °C for 1 h and cooled down to 0-5 °C. Next, 200 mL of MTBE was added into it, stired at 0-5 °C for 30min, filtered by suction, washed with MTBE, and dried in vacuum at 40 °C to obtain the product. ESI: m/z 763.40 $(M+H)^+$.

Preparation of Compound H: 0.57 g of **Compound D**, 0.34 g of p-toluenesulfonic acid monohydrate and 20 mL of acetonitrile were added into the reaction bottle, the mixture was reacted at 65 °C for 4 h, and evaporated to dryness under reduced pressure at 40 °C. Then, 20 ml of DMF, 0.3637 g of DIPEA, 0.1409 g of DCC and 0.0808 g of NHS were added into it, reacted at 35 °C for 15-20 h and cooled down to 25 °C. Next, 0.4341 g of intermediate G was added into it, the mixture was reacted for 1 h and evaporated to dryness under reduced pressure at 40 °C to obtain crude product, prepared by liquid phase method to obtain 66.5 mg of the target product. ESI: m/z 853.92 $(M+H+H)^{2+}/2$.

Preparation of Compound I: 66.5 mg of **Compound H**, 0.5 mL of piperidine

and 2 mL of DMF were added into the reaction bottle, the mixture was reacted at 25 °C for 1 h. Then, 10 mL of ethyl acetate was added dropwise into it to crystallize, the mixture was stirred for 30 min, filtered by suction, and the solid was dried in vacuum at 40 °C for 2 h to obtained 50.8 mg of the product. ESI: m/z 742.38 $(M+H+H)^{2+}/2$.

Preparation of Compound J: 39.5 mg of **Compound I**, 50.7 mg of intermediate Q, 10 mL of DIPEA and 2 mL of DMF were added into the reaction bottle, the mixture was reacted at 25 °C for 1 h, and evaporated to dryness under reduced pressure at 40 °C. Then, 2 mL of ethyl acetate and 2 mL of MTBE were added into it to crystallize, the mixture was stirred for 20 min, filtered by suction, and the solid was dried in vacuum at 40 °C, then obtained 43.2 mg of the product. ESI: m/z 941.52 $(M+H+H)^{2+}/2$.

Preparation of Compound K: 43.2 mg of **Compound J**, and 2 mL of trifluoroacetic acid were added into the reaction bottle, the mixture was reacted at 25 °C for 1 h, and evaporated to dryness under reduced pressure at 40 °C to obtain crude product. Then, it was purified with the prepared liquid phase method and lyophilized to obtain the product. ESI: m/z 884.95 $(M+H+H)^{2+}/2$.

The synthetic route is as follows:

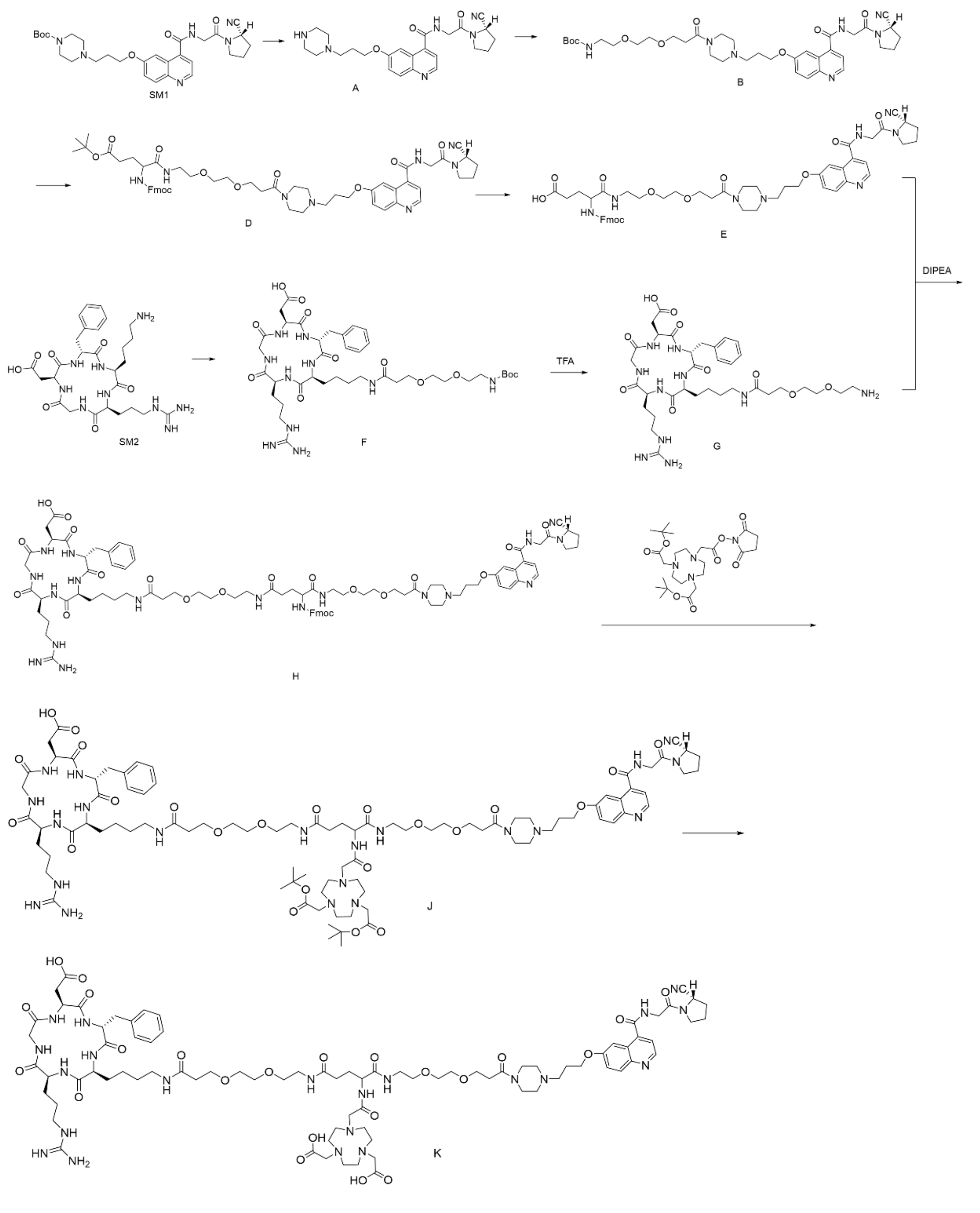

Synthetic route for NOTA-FAPI-RGD

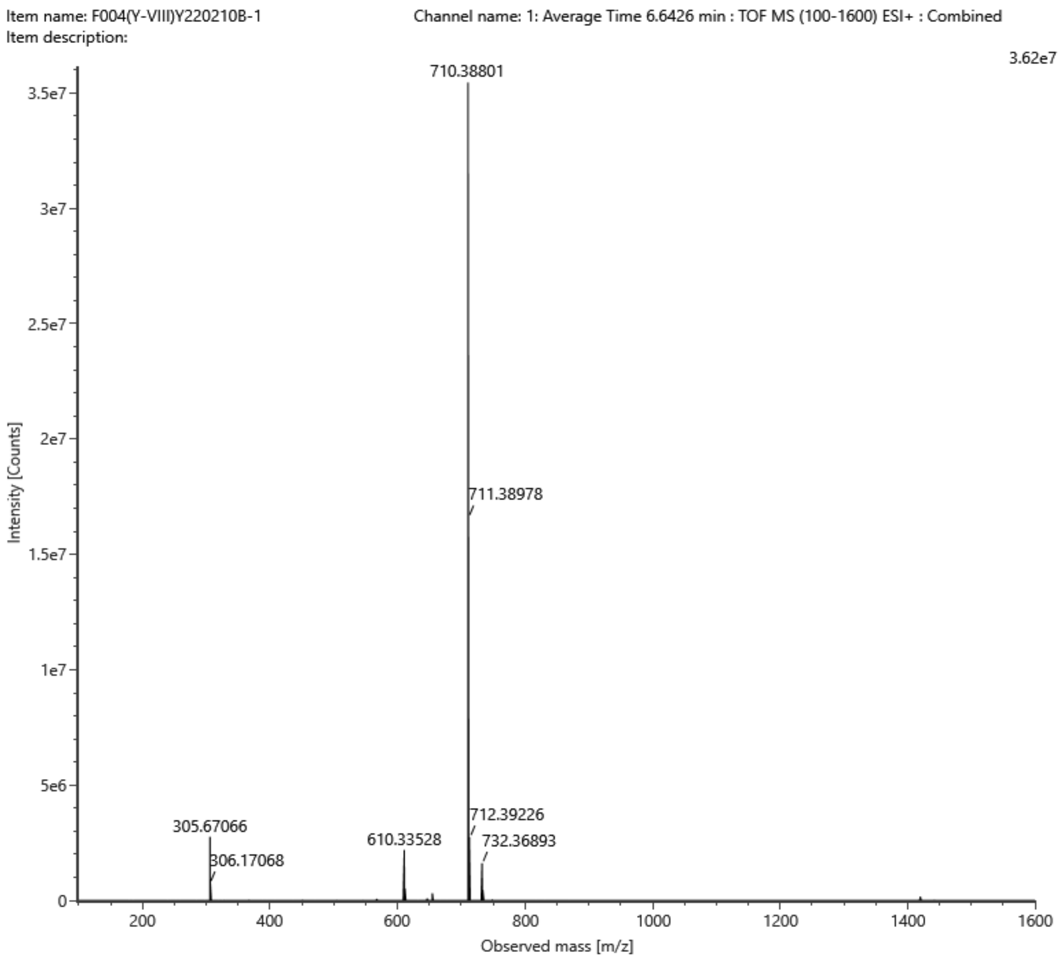

Figure S1. MS spectroscopy of compound B.

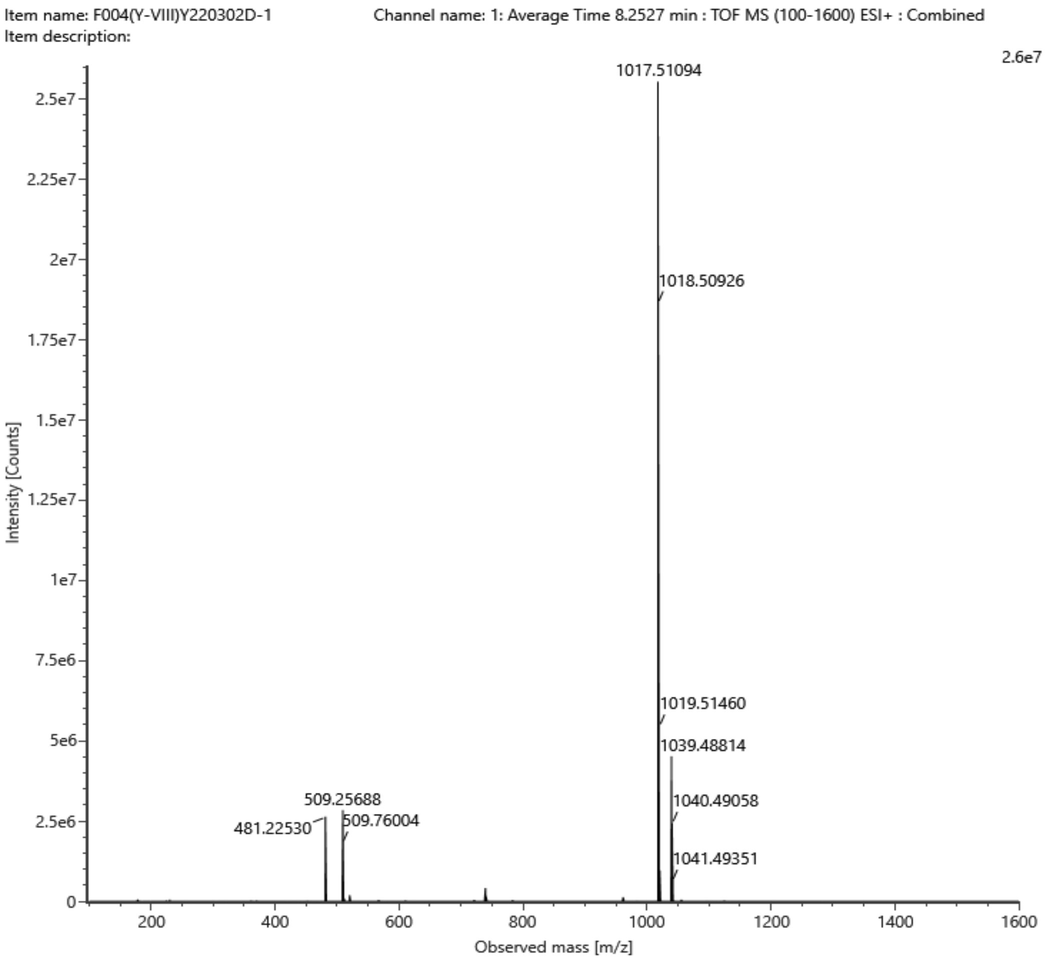

Figure S2. MS spectroscopy of compound D.

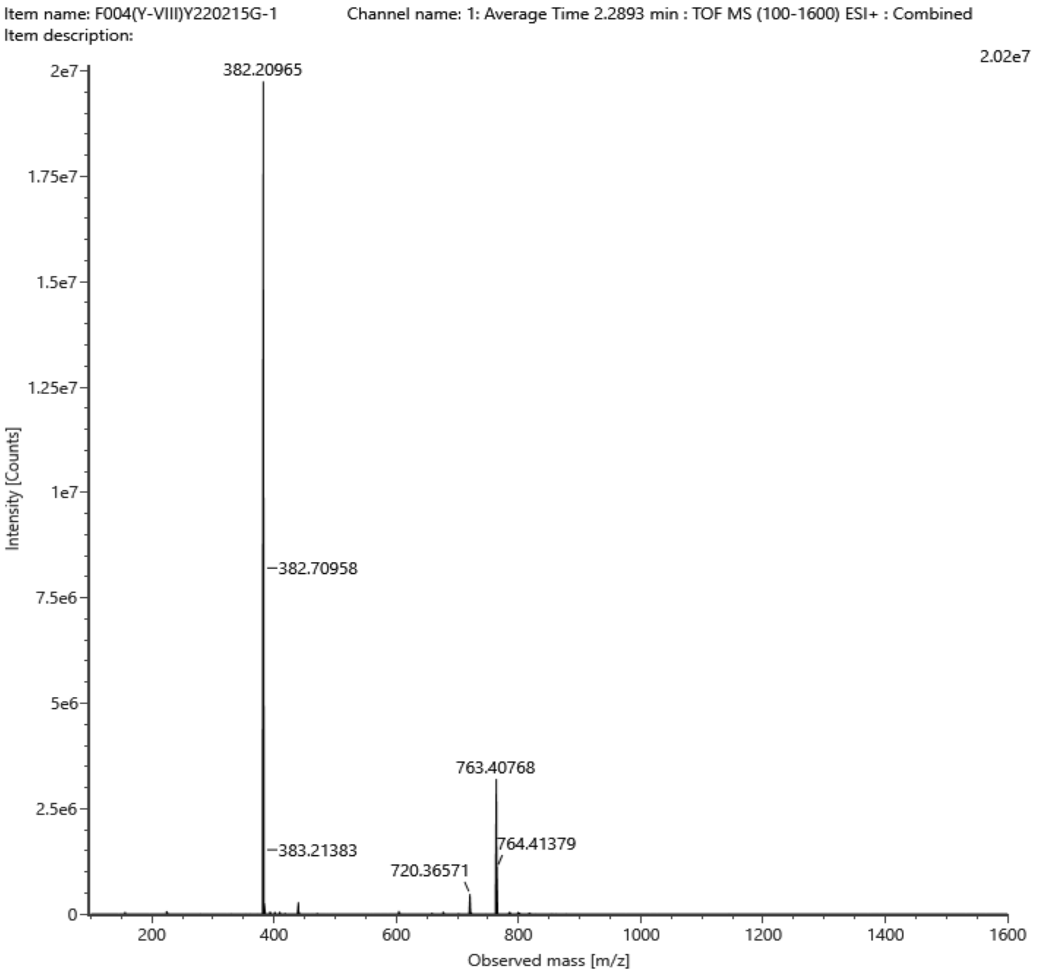

Figure S3. MS spectroscopy of compound G.

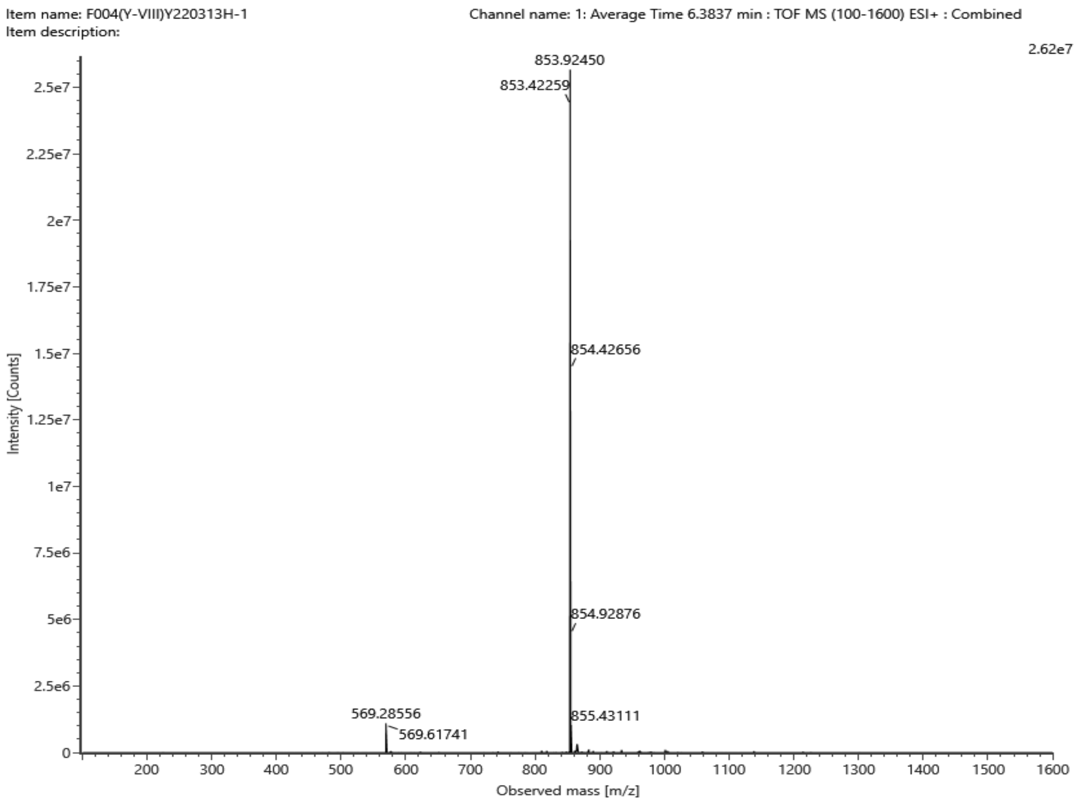

Figure S4. MS spectroscopy of compound H.

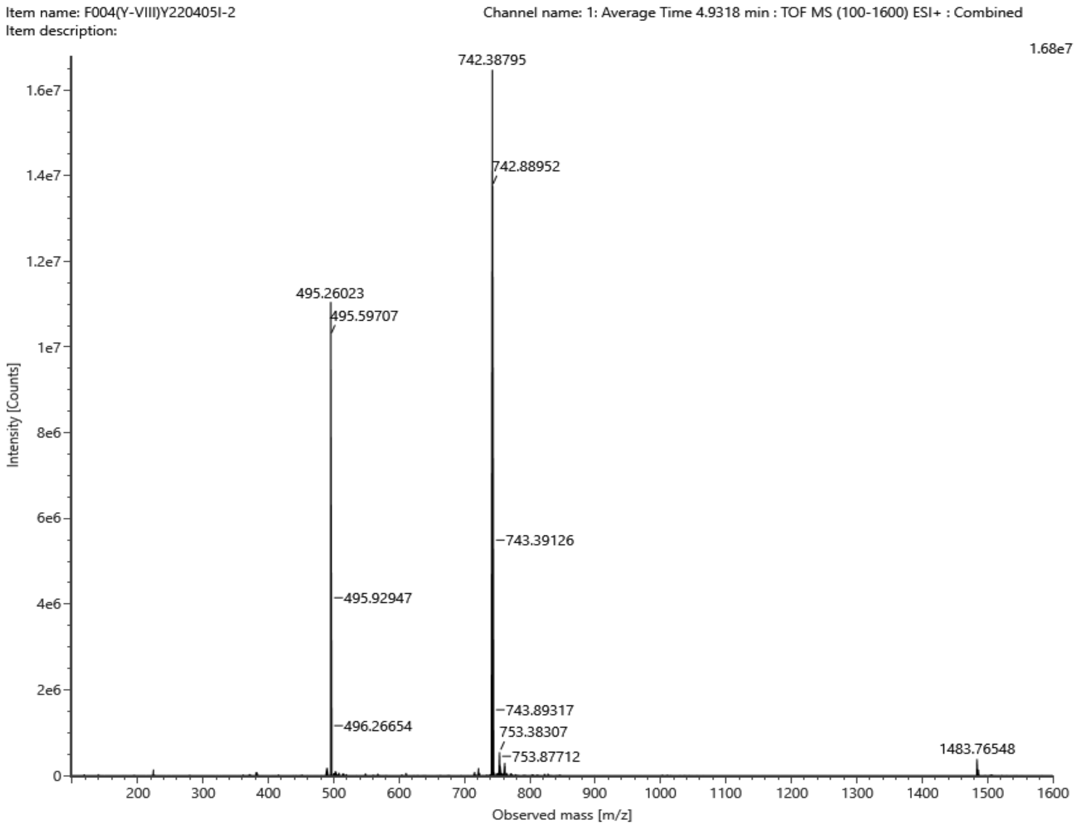

Figure S5. MS spectroscopy of compound I.

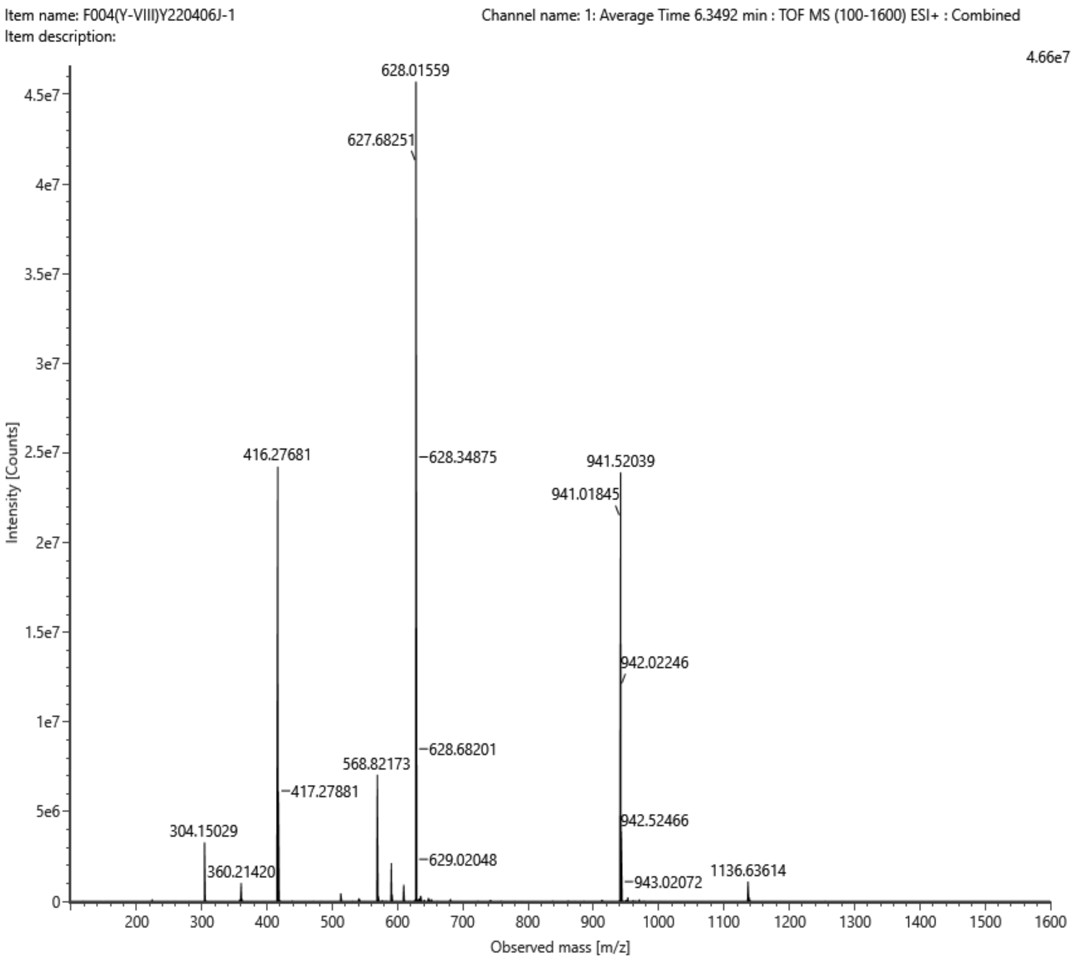

Figure S6. MS spectroscopy of compound J.

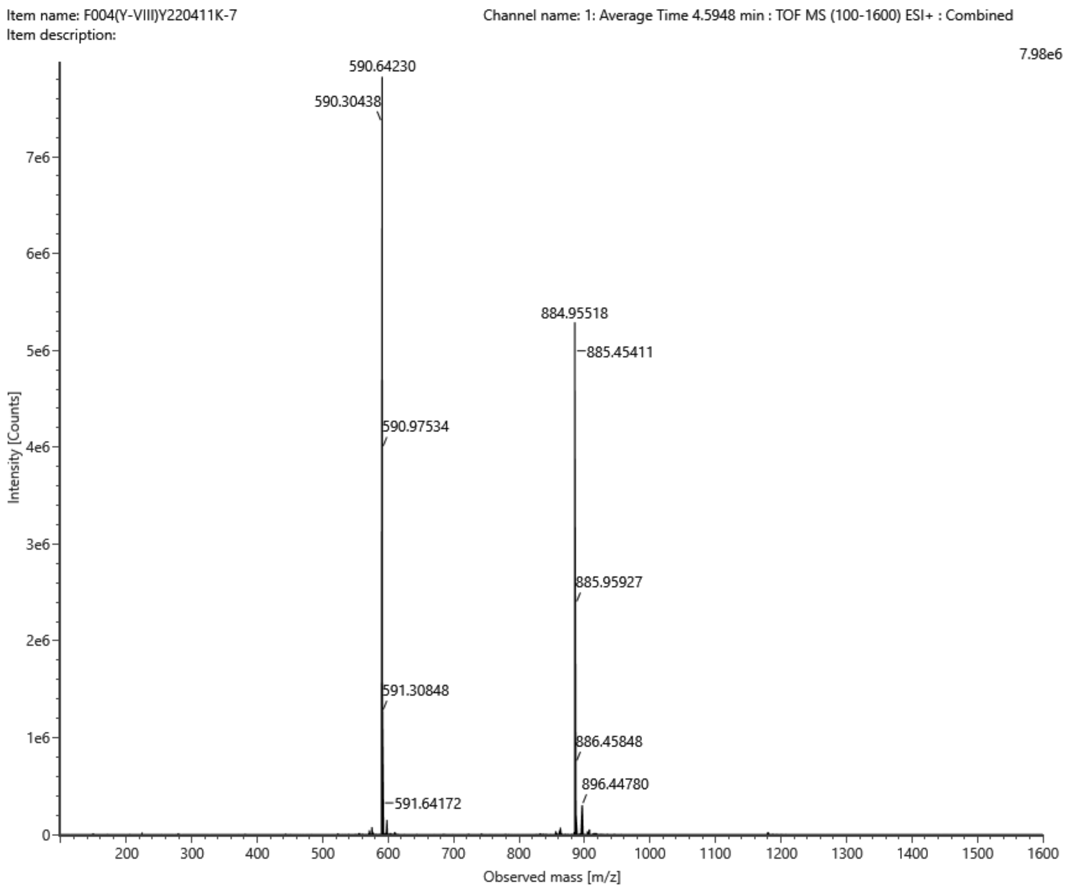

Figure S7. MS spectroscopy of compound NOTA-FAPI-RGD.

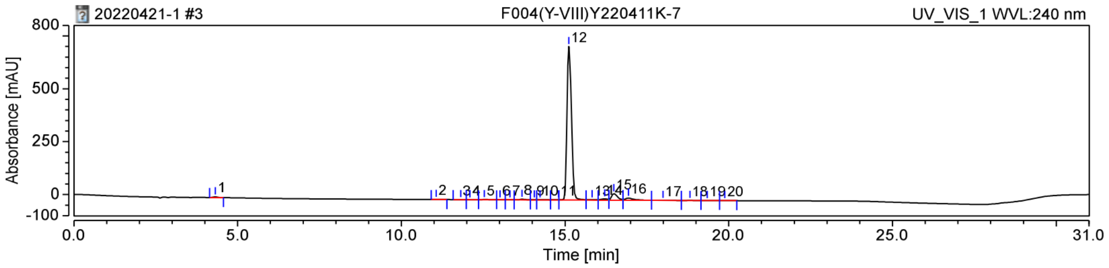

Figure S8. HPLC analysis of NOTA-FAPI-RGD.

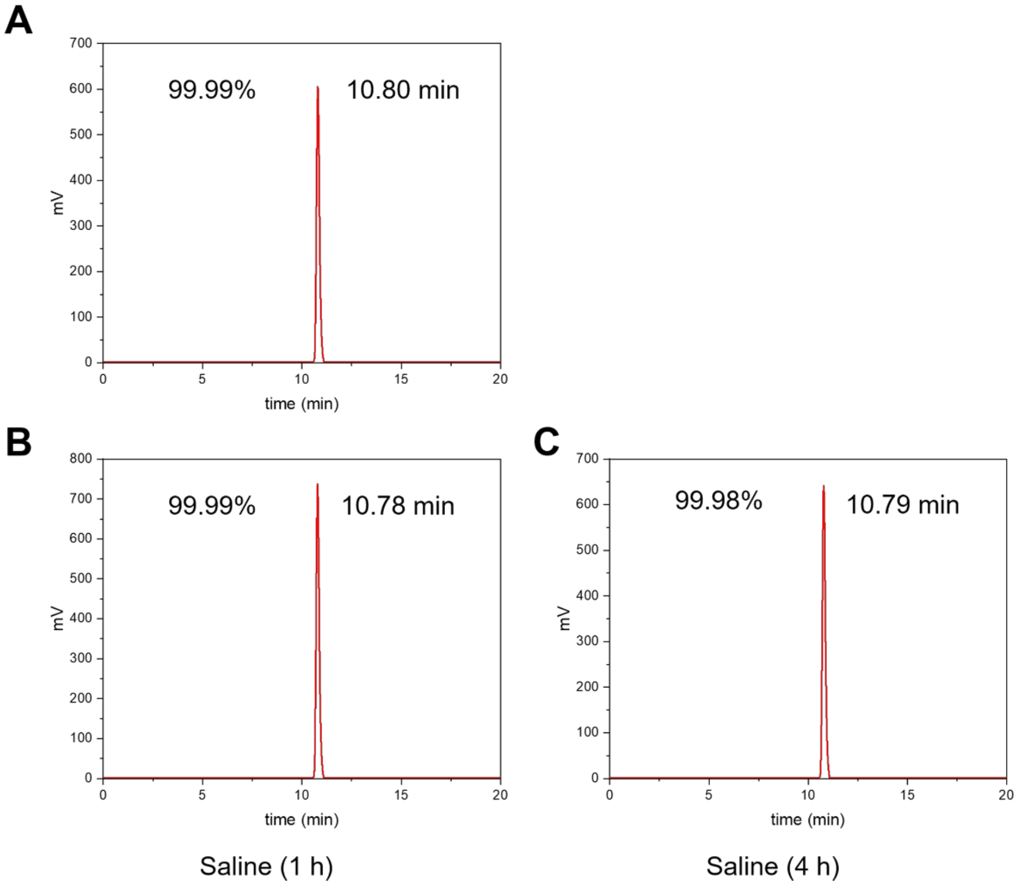

Figure S9. Radio-HPLC analysis of radiochemical purity (A) and stability (B, C) of [^{68}Ga]Ga-FAPI-RGD.

References

1. Wen X, Xu P, Shi M, et al. Evans blue-modified radiolabeled fibroblast activation protein inhibitor as long-acting cancer therapeutics. Theranostics. 2022,12(1): 422-433.
